# Portraying Mixed‐Agenda Couples: Insights From Large‐Scale Data Sets

**DOI:** 10.1111/jmft.70165

**Published:** 2026-08-21

**Authors:** Yike Tang, Shayne R. Anderson, Lee N. Johnson, Steven M. Harris, Anita Kateregga

**Affiliations:** ^1^ University of Minnesota Twin Cities Saint Paul Minnesota USA; ^2^ Brigham Young University Provo Utah USA

**Keywords:** couple therapy, discernment counseling, MFT‐PRN, mixed‐agenda couples, NCHAT, we‐ness

## Abstract

This study investigates “mixed‐agenda” couples characterized by significant discrepancies in commitment and therapy goals, using both clinical and non‐clinical data sets. While theoretical frameworks suggest these couples present unique challenges for treatment planning and therapy outcomes, empirical data on their prevalence have remained limited. We analyzed data from the National Couples' Health and Time Study (NCHAT) and the Marriage and Family Therapy Practice Research Network (MFT‐PRN) to identify groups of couples that might practically be classified as mixed‐agenda couples. Findings reveal that mixed‐agenda dynamics are prevalent, occurring in approximately 10% of the non‐clinical sample and nearly 40% of the clinical sample. This exceeds prior clinical estimates and confirms that a substantial portion of couples seeking therapy do so with conflicting motivations. These results validate the mixed‐agenda construct beyond anecdotal evidence, suggesting a need for tailored clinical interventions that address dyadic ambivalence to improve therapeutic efficacy and relationship stability.

## 1 Introduction

The term “mixed‐agenda couple” describes a relational configuration in which partners hold differing orientations toward the future of the relationship—typically one partner “leaning out” toward separation or divorce while the other “leaning in” toward maintaining the relationship or seeking relational rehabilitation (healing). Originally developed in the clinical work of Doherty and Harris (2016, 2017), this construct highlights a common crossroads presentation that complicates conventional conjoint couples therapy. Adjacent research on marital ambivalence, divorce ideation, and decision‐making processes indirectly supports the idea of mixed‐agenda dynamics, suggesting that these distressed relationships are often marked by commitment uncertainty, shifting thoughts about divorce, and competing orientations toward dissolution/reconciliation (Allen and Hawkins 2017; Hawkins et al. 2017; Joel et al. 2021). Although mixed‐agenda couples are recognized in therapeutic practice, empirical data directly documenting these couples remain limited.

Doherty et al. (2016) first introduced the term mixed‐agenda couples in their development of *Discernment Counseling*, an intervention designed specifically for couples in which partners disagree about whether to continue working on the relationship (Doherty and Harris 2017). In such dyads, the core assumption underlying couples therapy—mutual commitment to relationship improvement—does not hold. Comparable dynamics have been described in related literature using labels such as *polarized couples* (Abbasi 2017), *commitment uncertainty* (Allen et al. 2022; Owen et al. 2014), and *leaning‐in/leaning‐out couples* (Doherty et al. 2016). Although these terms emerge differently, they converge in describing a relational asymmetry in partners' motivation, emotional engagement, and interest in the future of the relationship.

Though direct prevalence studies of mixed‐agenda couples are scarce, several empirical strands lend support. First, national survey data show that a significant proportion of married individuals report ambivalence about their marriage. Hawkins et al. (2017), in a sample of approximately 3000 US married adults, found a substantial minority that reported recent thoughts about divorce or perceived their marriage to be in serious trouble. Hawkins and colleagues suggest that many marriages contain fluctuating commitment dynamics, making asymmetries at the partner level potentially common. Second, Harris et al. (2017) documented that individuals approaching divorce highly value clarity and confidence in their decision‐making, but find them to be elusive and fleeting, and often lead to ambivalence and conflicting motivations. Third, programmatic and clinic‐level data from Discernment Counseling initiatives (e.g., Emerson et al. 2020) show that couples presenting to such services frequently comprise partners with different degrees of commitment. While not nationally representative, these studies offer indirect evidence that mixed‐agenda patterns are prevalent in both clinical and non‐clinical contexts. Finally, Doherty and his colleagues (Doherty and Harris 2017; Doherty et al. 2011) have suggested that up to 30% of all couples that present for couples therapy may be mixed‐agenda couples.

Identifying mixed‐agenda couples matters for both contexts. Clinically, misclassification can lead to inappropriate treatment. Couples therapy assumes symmetrical motivation, with both individuals being similarly committed to working on the relationship. When one partner is leaning out, however, standard therapy can reinforce existing tensions or accelerate withdrawal. Differences in partners' investment in therapy can make it harder to establish shared goals and tasks for treatment. For example, when checking in with clients to see who has followed up on homework given in session, a leaning in partner can become discouraged if a leaning out partner consistently neglects or refuses to follow through on homework assignments designed to support the overall work and goals of therapy. This aspect of alliance has been conceptualized as the “within‐system alliance,” referring to partners' agreement on the nature of their difficulties, the goals of treatment, and the value of working together in therapy. Friedlander et al. (2018) identified this within‐system alliance as a central feature of conjoint treatment and found that split or unbalanced alliances were associated with poorer outcomes.

Discernment counseling addresses this by creating a structured decision‐making container, focusing on clarity about whether to pursue therapy, separate, or maintain the status quo. Research shows that this approach can reduce conflict and increase decision confidence (Emerson et al. 2020). Identifying mixed‐agenda status is equally important for accurate research design. Including (either knowingly or unwittingly) mixed‐agenda couples in clinical research without accounting for their unique motivational structure risks obscuring treatment effects and reducing internal validity.

Despite growing acknowledgment of the importance of mixed‐agenda couples, significant gaps remain. No large‐scale, population‐based studies have yet measured the prevalence of mixed‐agenda configurations by assessing both partners' orientations simultaneously. Additionally, standardized, psychometrically validated instruments to measure partner‐level lean‐in/lean‐out orientations have not been widely adopted. More rigorous intervention trials comparing discernment counseling with alternative approaches are also needed. To advance both clinical and scientific understanding of these couples, future research must establish baseline prevalence through nationally representative dyadic data sets and develop validated measures capable of identifying mixed‐agenda couples reliably.

In conclusion, mixed‐agenda couples represent a clinically significant and theoretically coherent relational configuration that affects treatment planning, intervention implementation, and family outcomes. While clinical evidence and related research point to the plausibility and importance of the construct, its prevalence remains supported primarily through indirect or anecdotal evidence. Larger‐scale, data‐driven studies are needed to validate and refine the mixed‐agenda concept.

The present study examines mixed‐agenda dynamics in both non‐clinical and clinical contexts. In the non‐clinical sample (Study One), we used partners' perceived likelihood of separation as a practical proxy for agenda alignment, which is the degree to which partners share a similar outlook on the future of the relationship. In the clinical sample (Study Two), we focused on four constructs from the clinical intake that capture dimensions reflecting partners' relational agendas. Commitment reflects each partner's investment in maintaining the relationship. We‐ness captures the extent to which partners perceive themselves as functioning as a team rather than as separate individuals (Gottman 1999). Hope reflects whether each partner believes the relationship can improve. Finally, pressure to attend therapy indicates whether one partner's participation in treatment is driven by external influence rather than internal motivation. Discrepancies or ambivalence between partners across these domains may signal fundamentally different agendas for the relationship and for therapy. Specifically, we aim to answer the following research questions: (1) What is the prevalence of couples with different agendas based on perceived separation likelihood in a non‐clinical sample (Study One)? (2) In a clinical sample, do couples cluster into distinct latent classes reflecting agenda alignment based on dyadic patterns of discrepancy or ambivalence in commitment, “we‐ness,” hope, and pressure to attend therapy (Study Two)? (3) How do couples identified as mixed‐agenda differ from other group(s) in relationship satisfaction and demographic/relationship characteristics, in both clinical and non‐clinical samples (Study One & Two)?

## 2 Method

As the idea of “mixed‐agenda” couples is a concept that has some anecdotal support but is lacking in empirical support, we decided to approach our inquiry with existing data sets. Because of this, neither of the studies we report here has specific measures of mixed‐agenda‐ness. So, we hope to approximate the dynamics associated with mixed‐agenda couple relationships with the variables we selected. The two studies draw on distinct samples that differ in recruitment context, measurement, and analytic purpose, and together provide complementary perspectives on mixed‐agenda dynamics. Study One uses a large, demographically diverse, non‐clinical sample (National Couples' Health and Time Study (NCHAT); Kamp Dush et al. 2023) composed of US couples recruited through population‐based survey methods, while Study Two uses a clinical sample drawn from the Marriage and Family Therapy Practice Research Network (MFT‐PRN; Johnson et al. 2017), consisting of couples presenting for therapy at university‐based clinics. Study One relies on a single indicator (i.e., perceived likelihood of separation) to estimate the prevalence of mixed‐agenda dynamics in the general population using descriptive analyses. Study Two employs multiple clinical indicators (i.e., commitment, we‐ness, hope, and pressure to attend therapy) and uses latent class analysis to identify empirically derived subgroups of couples based on patterns of dyadic discrepancy or ambivalence. Together, the two studies aim to move from a broad descriptive estimate of prevalence in the general population to a more nuanced, person‐centered classification of agenda alignment among couples actively seeking therapy.

### 2.1 Study One: Non‐Clinical

#### 2.1.1 Data and Sample

The non‐clinical data set, National Couples' Health and Time Study (NCHAT), is a comprehensive survey of US couples and provides a broad view of couple relational dynamics across a demographically diverse population (Kamp Dush et al. 2023). The data used in the current study are restricted use and available through the Inter‐university Consortium for Political and Social Research (ICPSR) Data Sharing for Demographic Research program (https://www.icpsr.umich.edu/web/DSDR/studies/38417). The full sample of Wave I included 3642 primary respondents and 1515 corresponding partners. In the current study, 28 cases were excluded due to missing data on our focal variable. Independent‐samples t‐tests comparing retained and excluded cases on several demographic variables (e.g., age, income, religiosity) were nonsignificant (all *p* > 0.05), suggesting no observable demographic bias associated with listwise deletion. After data cleaning and listwise deletion of cases with missing values, our final analytic sample included 1487 couples (2,974 individuals; 46.8% female, 48.9% male, 1% transgender, 3.2% other; 63.8% White, 8.3% Black, 13.4% Latinx, 7.1% Asian or Pacific Islander, 4.6% Multiracial, 1.2% Native American, and 1.6% identifying with another racial or ethnic group).

### 2.2 Measures

#### 2.2.1 Perceived Likelihood of Separation

Participants' perceived likelihood of separation was measured with a single item asking, “What do you think the chances are that you and your spouse/partner will eventually break up or separate?” Responses ranged from 1 (*very unlikely*) to 6 (*very likely*), with higher scores reflecting greater perceived risk of separation.

#### 2.2.2 Couple Relationship Satisfaction

Couple relationship satisfaction was assessed using the 4‐item Couple Satisfaction Index (CSI‐4; Funk and Rogge 2007). Items included “I have a warm and comfortable relationship with my spouse/partner,” “How rewarding is your relationship with your spouse/partner,” “In general, how committed are you to your current spouse/partner,” and “In general, how satisfied are you with your relationship?” Responses were rated on a 6‐point Likert scale ranging from 1 (*not at all)* to 6 (*completely*). A mean score was computed across the four items, with higher scores indicating greater relationship satisfaction. In the present sample, the scale demonstrated good internal consistency (*α* = 0.90).

#### 2.2.3 Data Analysis Plan

All analyses were conducted in R (v4.5.0). Partners were matched into dyads, and couples served as the unit of analysis. In NCHAT, because a direct measure of relationship agenda alignment was not available, we used partners' perceived likelihood of separation as a practical proxy. Responses of 1 (*very unlikely*) to 3 (*somewhat unlikely*) were coded as low separation likelihood, and responses of 4 (*somewhat likely*) to 6 (*very likely*) were coded as high separation likelihood. Couples were then classified as *aligned‐stay* if both partners reported low separation likelihood, *aligned‐leave* if both partners reported high separation likelihood, and *mixed‐agenda* if one partner reported low and the other reported high separation likelihood. After classifying, we calculated the percentage of couples in each group. Demographic and relationship characteristics, including age, education, income, relationship status, and relationship duration, were summarized using means and standard deviations for continuous variables and proportions for categorical variables (see Table 1). One‐way ANOVAs were then conducted to test differences in relationship satisfaction across the three agenda groups.

**Table 1:** Demographics of respondents by relationship agenda alignment.

	Aligned‐stay	Aligned‐leave	Mixed‐agenda	Lean‐in	Lean‐out
Percentage	86.42	3.70	9.89	\	\
Age	42.59 (10.87)	40.42 (9.39)	41.48 (10.56)	42.09 (11.00)	40.88 (10.11)
Education level					
GED/high school or less	372 (14.47%)	17 (15.45%)	45 (15.31%)	24 (16.33%)	21 (14.29%)
Vocational/technical/associate	693 (26.96%)	32 (29.09%)	96 (32.65%)	55 (37.41%)	41 (27.89%)
Bachelor's degree	760 (29.57%)	38 (34.55%)	81 (27.55%)	38 (25.85%)	43 (29.25%)
Graduate/professional degree	730 (28.40%)	22 (20.00%)	65 (22.10%)	27 (17.68%)	39 (26.53%)
Income (k)	66.63 (59.02)	55.76 (55.95)	55.01 (45.68)	53.35 (42.65)	61.66 (73.64)
Relationship status					
Legal marriage	837 (65.14%)	23 (41.82%)	72 (48.98%)	\	\
Commitment ceremony	78 (6.07%)	2 (3.64%)	5 (3.40%)	\	\
Registered domestic partnership/Civil union	96 (7.47%)	3 (5.45%)	12 (8.16%)	\	\
Other	271 (21.09%)	26 (47.27%)	57 (38.78%)	\	\
Relationship length	16.55 (9.82)	14.03 (8.92)	13.99 (9.66)	\	\
Satisfaction (1–6)	5.24 (0.76)	3.03 (1.03)	4.18 (1.06)	4.67 (0.85)	3.69 (1.03)

*Note:* Values are presented as *M* (SD) for continuous variables and *n* (%) for categorical variables. Income contained extreme values; values below the first and above the 99th percentile were deleted before computing summary statistics.

### 2.3 Study Two: Clinical

#### 2.3.1 Data and Sample

The clinical data set comes from the Marriage and Family Therapy Practice Research Network (MFT‐PRN) and consists of routine clinical assessments collected from a variety of couple therapy cases at participating university‐based family therapy clinics across the United States (Johnson et al. 2017). The data used in the current study contains protected clinical information and is not publicly available; inquiries regarding data access may be directed to the MFT‐PRN (https://www.mft-prn.net). This study used assessment data collected at the first therapy session and included only clients who presented for couples therapy.

After initial data preparation, we restricted the data set to Session 1 and retained only cases with data from both partners. We then constructed couple‐level indicators for the latent class analysis based on the two partners' responses. To evaluate the missingness, we conducted Little's MCAR test using the focal LCA indicators. The test was nonsignificant, *χ^2^* (5) = 10.30, *p* = 0.066, suggesting that the missingness pattern did not significantly deviate from missing completely at random. Therefore, we deleted cases with missing data listwise. After data cleaning, 913 couples (1,826 individuals) were included in the analysis (50.2% female, 48.8% male, 0.2% transgender, 0.7% other; 80.5% White, 2.5% Black, 5.9% Latinx, 2.4% Asian, 7.7% Multiracial, and 1% identifying with another racial or ethnic group).

### 2.4 Measures

#### 2.4.1 Commitment Discrepancy

Commitment to the relationship was measured with a single item from the Couple Relationship Scale (Anderson et al. 2022). Using the stem “Describe how you feel about your relationship with your partner over the past week,” participants rated how they felt on a visual analog scale ranging from 0 (*like giving up*) to 100 (*completely committed*). The CRS has demonstrated strong concurrent and construct validity and good internal consistency (Anderson et al. 2022). Couples were coded as showing *commitment discrepancy* when one partner scored below the scale midpoint (50) and the other scored above, and partners differed by more than 16 points. This threshold was based on the reliable change index (RCI) for the full CRS scale.

#### 2.4.2 “We‐Ness” Discrepancy or Ambivalence

*We‐ness* was assessed using a single item: “I really feel like part of a team with my partner” from the Couple Satisfaction Index (CSI‐16; Funk and Rogge 2007). Responses were recorded on a 6‐point Likert scale ranging from 0 (*not at all true*) to 5 (*completely true*). Couples were coded as showing *we‐ness discrepancy or ambivalence* if one partner scored below 2 and the other above 3, or both partners scored between 2 and 3.

#### 2.4.3 Hope Discrepancy or Ambivalence

Hope for the relationship was measured with a single item, “Select the answer that best describes how you feel about your relationship,” from the Couple Satisfaction Index (CSI‐16; Funk and Rogge 2007), with responses ranging from 0 (*discouraging*) to 5 (*hopeful*). Couples were coded as showing *hope discrepancy or ambivalence* if one partner rated hope below 2 and the other above 3, or both partners rated hope between 2 and 3.

#### 2.4.4 Pressure to Attend Therapy Discrepancy

Perceived pressure to attend therapy was measured with a single item, “How much did someone else pressure you to come to therapy?” Responses ranged from 0 (*not at all*) to 5 (*very pressured*). Couples were coded as showing *pressure discrepancy* when one partner reported no pressure (score of 0), and the other reported a score above 0, reflecting some degree of perceived pressure.

#### 2.4.5 Couple Relationship Satisfaction

Relationship satisfaction was measured using the 16‐item Couple Satisfaction Index (CSI‐16; Funk and Rogge 2007). Sample items include “Please indicate the degree of happiness, all things considered, of your relationship.” “In general, how often do you think that things between you and your partner are going well?” and “Our relationship is strong.” Responses were averaged across the 16 items to create a composite mean score, with higher scores reflecting greater relationship satisfaction. Internal consistency in the present sample was excellent (*α* = 0.98).

#### 2.4.6 Data Analysis Plan

Latent class analysis (LCA) was first conducted to identify whether couples could be grouped into distinct patterns of relationship agenda alignment based on four observed categorical indicators: commitment discrepancy or ambivalence, we‐ness discrepancy or ambivalence, hope discrepancy or ambivalence, and pressure to attend therapy discrepancy. LCA is a person‐centered method that identifies unobserved subgroups based on response patterns across observed variables (Collins and Lanza 2013). It assumes conditional independence, which means that the observed indicators are assumed to be independent within each latent class, with associations among the indicators fully explained by class membership. To determine the optimal number of latent classes, we estimated a series of models beginning with a one‐class solution and sequentially increasing the number of classes. Model selection followed an exploratory process guided by multiple fit indices (Nylund‐Gibson and Choi 2018): (1) the Akaike Information Criterion (AIC); (2) the Bayesian Information Criterion (BIC); (3) entropy; and (4) Bootstrap Likelihood Ratio Test (BLRT). Lower AIC and BIC values both indicate better model fit (Nylund‐Gibson and Choi 2018). Entropy reflects the precision of class assignment, with values closer to 1 indicating clearer classification. BLRT compares the *k*‐class model to the (*k*−1)‐class model, with a statistically significant result (*p* < 0.05), indicating that the model with *k* classes provides improved fit (Nylund et al. 2007). All LCA models were estimated using maximum likelihood estimation. To reduce the risk of converging on local maxima, each model was estimated using 100 sets of random starting values, with the 20 best solutions retained for final‐stage optimization. Convergence was evaluated based on the change in log‐likelihood between iterations (ΔLog‐Likelihood ≤ 0.0001). A reproducibility seed was set prior to estimation (set.seed = 123).

In this study, partners were analyzed as dyads, with couples serving as the unit of analysis. Latent class analysis and the Bootstrap Likelihood Ratio Test were conducted in R (v4.5.0) using the *LCPA* package (Qin and Guo 2026). Descriptive statistics and independent‐samples *t* tests were computed using base R. To parallel the descriptive analyses in the NCHAT sample, demographic and relationship characteristics were summarized by latent class. Because the LCA supported a two‐class solution, independent‐samples *t* tests were used to compare classes on couples' relationship satisfaction.

## 3 Results

### 3.1 Study One: Non‐Clinical Sample Nchat

#### 3.1.1 General Characteristics by Group

After categorization, 147 couples (9.89%) were identified as mixed‐agenda, meaning one partner reported low separation likelihood while the other reported high separation likelihood; 1,285 (86.42%) as aligned‐stay, meaning both partners reported low perceived likelihood of separation; and 55 (3.70%) as aligned‐leave, meaning both partners reported high perceived likelihood of separation. Table 1 presents the demographic and relationship characteristics of the sample by relationship agenda group. One‐way analyses of variance (ANOVAs) were conducted comparing couples on couple‐level mean relationship satisfaction. Results indicated significant group differences in relationship satisfaction, *F* (2, 1468) = 412.30, *p* < 0.001. Tukey post hoc tests showed that all pairwise comparisons were significant. Relationship satisfaction was highest among aligned‐stay couples, followed by mixed‐agenda couples, and lowest among aligned‐leave couples.

### 3.2 Study Two: Clinical MFT‐PRN Sample

#### 3.2.1 Latent Class Analysis

As shown in Table 2, the fit indices supported retention of the two‐class solution. The two‐class model had the lowest AIC and BIC values, indicating the best relative model fit among the estimated models. The entropy value for the two‐class solution (0.715) suggested moderate classification precision and indicated clearer class separation than the three‐ and four‐class solutions. Additionally, the BLRT suggested that the three‐class model did not significantly improve fit over the two‐class model (*p* = 0.539 > 0.05). Furthermore, the two‐class solution demonstrated stable estimation, with the best log‐likelihood value successfully replicated across the final‐stage random starts, suggesting that the model was unlikely to reflect a local maximum. Both classes were of sufficient size to support stable parameter estimation (Class 1: *n* = 545, 59.7%; Class 2: *n* = 368, 40.3%). Beyond statistical fit, the two‐class solution was retained on substantive grounds. The two classes were theoretically interpretable and clinically relevant, distinguishing couples who entered therapy with mixed agendas from those who presented with broadly aligned relational agendas. Models with additional classes did not produce clearly differentiated subgroups beyond this distinction. Taken together, all fit indices converged in supporting the two‐class solution. Accordingly, we selected the two‐class solution as the final model.

**Table 2:** Fitting indices of LCA models with one to four classes.

Classes	AIC	BIC	Entropy	BLRT (*p*‐value)
1	4536.389	4555.656	/	/
2	4358.669	4421.287	0.715	0.000***
3	4372.738	4478.706	0.713	0.539
4	4390.236	4539.554	0.448	0.828

***p < 0.001.

#### 3.2.2 Class Interpretation

**Figure 1:**
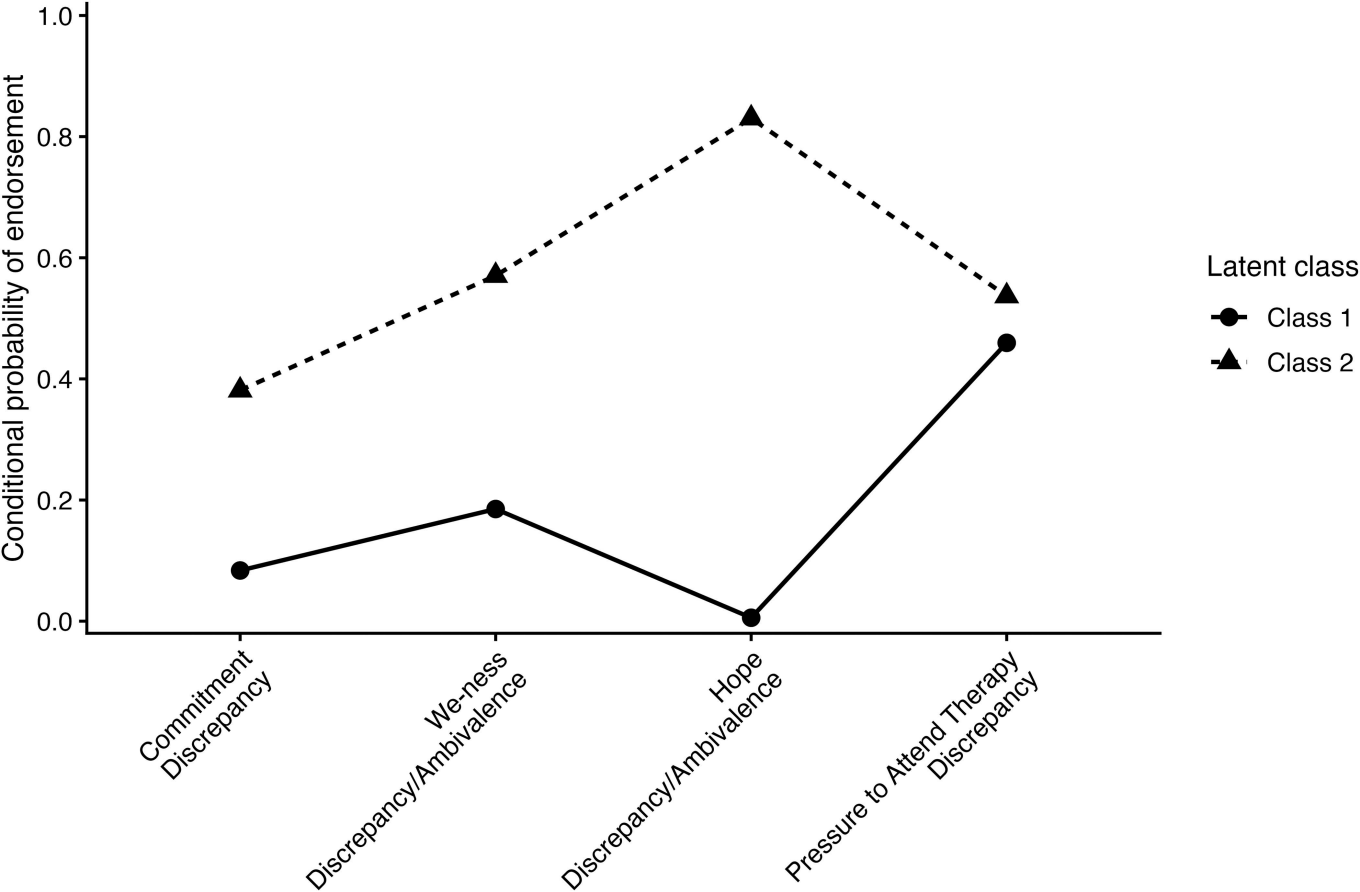
Conditional item probabilities for the two‐class latent class solution.

Figure 1 shows the conditional item probabilities for the two‐class solution. In latent class analysis, a conditional item probability represents the likelihood that an individual is assigned to a given class according to a particular indicator. In the current study, higher values reflect a greater likelihood of couple‐level mismatch or ambivalence in that domain. The pattern of conditional probabilities across indicators defines and differentiates the latent classes.

Class 1 comprised 1090 individuals (545 couples; 59.7%) and was labeled the *aligned couple* class. This class showed low probabilities of discrepancy or ambivalence in commitment (8.4%), we‐ness (18.5%), and hope (0.6%), suggesting that couples in this class generally entered therapy with relatively shared relational investment, sense of couple unity, and feelings of hope toward the relationship. This class also presented a modest probability of discrepancy in pressure to attend therapy (46.0%), indicating that even among otherwise aligned couples, partners did not always enter therapy with the same level of perceived pressure.

Class 2 comprised 736 individuals (368 couples; 40.3%) and was labeled the *mixed‐agenda couple* class. In contrast, this class showed higher probabilities of discrepancy or ambivalence in commitment (38.1%), we‐ness (57.0%), and hope (83.0%). The very high probability of hope discrepancy or ambivalence was the most distinctive feature of this class, suggesting that partners in this group were particularly likely to differ in, or feel uncertain about, whether the relationship is hopeful. This class also showed a slightly higher probability of discrepancy in pressure to attend therapy (53.7%). Taken together, this pattern supports the mixed‐agenda label that couples in this class appeared less consistently aligned around relational commitment, couple identity, and hope for the relationship, even though pressure to attend therapy differentiated the classes less strongly than the other indicators.

#### 3.2.3 General Characteristics by Class

To further interpret the two‐class solution, Table 3 summarizes demographic and relationship characteristics by latent class membership. To parallel the analyses conducted in the NCHAT sample, independent‐samples *t* tests were used to compare the two classes on relationship satisfaction. Participants in the mixed‐agenda couple class reported significantly lower satisfaction (*M* = 43.44) than those in the aligned class (*M* = 61.37), Welch's *t* (1573.7) = 24.47, *p* < 0.001.

**Table 3:** General characteristics of the sample by mixed‐agenda classes.

	Aligned (*N* = 1090)	Mixed‐agenda (*N* = 736)
Percentage	59.7	40.3
Age	28.3 (8.57)	32.2 (9.96)
Education level		
GED/high school or less	336 (28.89%)	170 (25.68%)
Vocational/technical/associate	279 (23.97%)	143 (21.60%)
Bachelor's degree	389 (33.4%)	216 (32.6%)
Graduate/professional degree	160 (13.8%)	133 (20.1%)
Household Income (k)	55.27 (47.80)	64.70 (48.24)
Marital status		
Married	749 (64.4%)	468 (70.7%)
Divorced	32 (2.75%)	26 (3.93%)
Separated	10 (0.86%)	6 (0.91%)
Remarried	10 (0.86%)	6 (0.91%)
Relationship length	4.70 (6.52)	7.69 (8.14)
Satisfaction (0–100)	61.4 (16.7)	43.4 (14.0)

*Note:* Values are presented as *M* (SD) for continuous variables and *n* (%) for categorical variables.

## 4 Discussion

At the outset of this project, we were attempting to see if we could identify, beyond anecdotal accounts, the existence of mixed‐agenda couples in both clinical and non‐clinical samples. What we discovered was a sizeable difference in these couples by sample type. We found about 10% mixed‐agenda couples in the non‐clinical sample and almost 40% of these couples in the clinical sample. The clinical number is higher than estimates made by Doherty and Harris (2017), who suggested 25%–30% of all couples seeking couples therapy are likely to be mixed‐agenda couples. The results of the current analysis seem to support the idea that mixed‐agenda couples present often or regularly in couples therapy. This also supports what Doss et al. (2004) have suggested earlier, “…that when an individual couple presents for marital therapy, the [individual partners] are likely doing it for very different reasons” (p. 611). Thus, the current study's two‐class solution supports the idea that in a real‐world therapy situation, couples can be sorted into “aligned” and “mixed‐agenda” groupings, thereby supporting the “discovery” of mixed‐agenda couples beyond hearsay evidence. The specific variables used in this study were commitment (to the relationship), we‐ness (sense of team), hopefulness (regarding the relationship), and feeling pressured to attend therapy. Because standard couple therapy presumes shared goals, identifying mixed‐agenda couples early may help clinicians decide what approach is most appropriate; however, these clinical suggestions are preliminary and require empirical testing. Future research might explore whether launching into some form of couples therapy or having the couple begin with discernment counseling would help mixed‐agenda couples get aligned in purpose first.

The non‐clinical sample number of 10% mixed‐agenda couples is really a first attempt to identify these couples in a large‐scale data set. We anticipate that more research in the future will verify or provide greater clarity on the amount of mixed‐agenda “ness” that occurs in typical, non‐clinical couple relationships. Further, the implications for couple health or marital satisfaction would seem to be connected to how much a couple experiences similar levels of “we‐ness” or alignment across key dimensions (Gottman 1999). Gottman has suggested that the construct of “we‐ness” is paramount to marital and relational health, personal well‐being, and relationship longevity.

The strengths of the current study include seeking to empirically validate the existence of mixed‐agenda couples with two large‐scale data sets. A further benefit is that one data set was generated as part of a clinical process, and the other was a more population‐representative data set. Having access to both types of data can provide a well‐rounded picture of the phenomenon in question. Concerning mixed‐agenda couples that present in clinical settings, it is paramount to clinical success to identify mixed‐agenda couples early so that therapy can be as successful as possible. If discrepant agendas are not recognized at the outset, therapists may proceed as though both partners are similarly invested in the relationship and in therapy, when this is not actually the case. Prior research suggests that early discontinuance is tied to therapeutic alliance (Anderson et al. 2019), and pressure to attend therapy is associated with poorer alliance formation (Anderson et al. 2020). Viewed together with the present findings, these patterns suggest a possible connection between mixed‐agenda status and premature disengagement or limited benefit from treatment, potentially through links with other therapeutic processes, such as the therapeutic alliance. Because the present study did not directly examine dropout or treatment outcome, this interpretation remains speculative. Still, it may be worth considering whether some couples who discontinue therapy early, or who do not improve, could be mixed‐agenda couples whose differing levels of commitment and therapy investment were not adequately identified or addressed. By contrast, there may be less of an urgency to identify mixed‐agenda couples that are not presenting for clinical intervention, although recognizing this pattern may still be valuable for understanding relationship trajectories outside of therapy.

One major limitation of the present study is the reliance on secondary data, which limited the operational definition of “mixed‐agenda” couple. Ideally, mixed‐agenda couple status would be defined using a more comprehensive assessment of partners' relational intentions and commitment processes. However, in the NCHAT data, classification relied primarily on a single item assessing separation likelihood. As a result, the identified group could not be interpreted as a direct representation of mixed‐agenda couples as they may be conceptualized theoretically. Meanwhile, the PRN classification was derived from patterns of discrepancy and ambivalence across multiple variables rather than from a direct measure of mixed‐agenda dynamics. This approach examined the latent relationship uncertainty, but it also means that prevalence estimates are partly shaped by the available indicators and measurement structure. For example, MFT‐PRN indicators were based on single items. Because these indicators were drawn from secondary data sources, reliability and validity evidence for these specific operationalizations was not available. Although single‐item indicators may improve clinical interpretability, they may also fail to capture the full complexity of the underlying constructs. Accordingly, the observed prevalence of “mixed‐agenda couple” should also be interpreted as suggestive rather than definitive. Related to the secondary data limitation is the fact that we were unable to control for distress levels between the two samples. The clinical sample may show more mixed‐agenda couples due to the higher levels of distress typical of clinical populations. However, we were unable to declare this with any confidence.

While this first attempt at identifying mixed‐agenda couples empirically is an important step in this research, future research in this area should include clinical assessments with measures specifically designed to assess for mixed‐agenda couples. Doherty and Harris (2017) offer one such scaling instrument that may have clinical utility (see Appendix SA). Using this instrument in clinical work with all couples could help identify and sort clinical couples to the correct level of relational interventions. We are hopeful that the inclusion of this instrument in the current study will shape future studies in this area. Additionally, it would be important for the appropriate norming and psychometric research to be conducted on this instrument to establish its reliability and validity. Finally, having a greater understanding of mixed‐agenda couples among clinical and non‐clinical samples can help us understand their relationship to other concepts, such as personal well‐being, co‐parent cooperation, and general life satisfaction as well.

## 5 Conclusion

Mixed‐agenda couples have long been recognized in clinical practice, yet empirical evidence documenting their presence and distribution across settings has remained limited. The present study extends this literature by examining mixed‐agenda dynamics from both a non‐clinical, population‐based sample and a clinical couples therapy sample. Our findings suggested that mixed‐agenda dynamics might be more common in clinical settings, and in both contexts were associated with lower relationship satisfaction than more aligned couples. Taken together, these findings suggest that it may be important to assess couple alignment more directly, rather than assuming that partners come to therapy with similar levels of commitment, hope, or readiness to work on the relationship. However, this study should be understood as an early step as both data sets relied on secondary indicators. Even so, the findings point to the value of developing stronger measures, and future research could continue to study how mixed‐agenda dynamics shape therapy engagement, alliance, dropout, and treatment outcomes over time.

## Funding

The authors have nothing to report.

## Supporting information


Supporting File 1

## Data Availability

The first data that support the findings of this study are available from the NCHAT team. Restrictions apply to the availability of these data, which were used under license for this study. Data are available from https://www.icpsr.umich.edu/web/DSDR/studies/38417 with application. The second data that support the findings of this study are available from MFT‐PRN. Restrictions apply to the availability of these data, which were used under license for this study. Data are available from https://www.mft-prn.net by contacting contact@mft-prn.com.

## References

[jmft70165-bib-0001] Abbasi, I. S. 2017. “Polarized Couples in Therapy: Recognizing Indifference as the Opposite of Love.” Journal of Family Psychotherapy 28, no. 1: 52–67. 10.1080/08975353.2017.1282395. 26684280

[jmft70165-bib-0002] Allen, S. , and A. J. Hawkins . 2017. “Theorizing the Decision‐Making Process for Divorce or Reconciliation.” Journal of Family Theory & Review 9, no. 1: 50–68. 10.1111/jftr.12176.

[jmft70165-bib-0003] Allen, S. , A. J. Hawkins , S. M. Harris , K. Roberts , A. Hubbard , and M. Doman . 2022. “Day‐to‐Day Changes and Longer‐Term Adjustments to Divorce Ideation: Marital Commitment Uncertainty Processes Over Time.” Family Relations 71, no. 2: 611–629. 10.1111/fare.12599.

[jmft70165-bib-0004] Anderson, S. R. , A. Banford Witting , R. R. Tambling , S. A. Ketring , and L. N. Johnson . 2020. “Pressure to Attend Therapy, Dyadic Adjustment, and Adverse Childhood Experiences: Direct and Indirect Effects on the Therapeutic Alliance in Couples Therapy.” Journal of Marital and Family Therapy 46, no. 2: 366–380. 10.1111/jmft.12394. 31219191

[jmft70165-bib-0005] Anderson, S. R. , L. N. Johnson , R. B. Miller , and C. C. Barham . 2022. “The Couple Relationship Scale: A Brief Measure to Facilitate Routine Outcome Monitoring in Couple Therapy.” Journal of Marital and Family Therapy 48, no. 2: 464–483. 10.1111/jmft.12541. 34269484

[jmft70165-bib-0006] Anderson, S. R. , R. Tambling , J. B. Yorgason , and E. Rackham . 2019. “The Mediating Role of the Therapeutic Alliance in Understanding Early Discontinuance.” Psychotherapy Research 29, no. 7: 882–893. 10.1080/10503307.2018.1506949. 30079816

[jmft70165-bib-0007] Collins, L. M. , and S. T. Lanza . 2013. Latent Class and Latent Transition Analysis: With Applications in the Social, Behavioral, and Health Sciences. John Wiley & Sons.

[jmft70165-bib-0008] Doherty, W. J. , and S. M. Harris . 2017. Helping Couples on the Brink of Divorce: Discernment Counseling for Troubled Relationships. American Psychological Association.

[jmft70165-bib-0009] Doherty, W. J. , S. M. Harris , and J. L. Wilde . 2016. “Discernment Counseling for “Mixed‐Agenda” Couples.” Journal of Marital and Family Therapy 42, no. 3: 246–255. 10.1111/jmft.12175. 26189438

[jmft70165-bib-0010] Doherty, W. J. , B. J. Willoughby , and B. Peterson . 2011. “Interest in Marital Reconciliation Among Divorcing Parents: Interest in Marital Reconciliation Among Divorcing Parents.” Family Court Review 49: 313–321. 10.1111/j.1744-1617.2011.01373.x.

[jmft70165-bib-0011] Doss, B. D. , L. E. Simpson , and A. Christensen . 2004. “Why Do Couples Seek Marital Therapy?” Professional Psychology: Research and Practice 35, no. 6: 608–614. 10.1037/0735-7028.35.6.608.

[jmft70165-bib-0012] Emerson, A. J. , S. M. Harris , and K. Reece . 2020. “The Impact of Discernment Counseling on Individuals Considering Divorce.” Journal of Marital and Family Therapy 46, no. 2: 317–332. 10.1111/jmft.12413. PMC7894569 33159369

[jmft70165-bib-0024] Friedlander, M. L. , V. Escudero , M. J. Welmers‐van de Poll , and L. Heatherington . 2018. “Meta‐Analysis of the Alliance–Outcome Relation in Couple and Family Therapy.” Psychotherapy 55, no. 4: 356–371. 10.1037/pst0000161. 30335450

[jmft70165-bib-0013] Funk, J. L. , and R. D. Rogge . 2007. *Couples Satisfaction Index (CSI)* [Database record]. APA PsycTests. 10.1037/t01850-000.

[jmft70165-bib-0014] Gottman, J. M. 1999. The Marriage Clinic: A Scientifically Based Marital Therapy. WW Norton & Company.

[jmft70165-bib-0015] Harris, S. M. , S. A. Crabtree , N. K. Bell , S. M. Allen , and K. M. Roberts . 2017. “Seeking Clarity and Confidence in the Divorce Decision‐Making Process.” Journal of Divorce & Remarriage 58, no. 3: 186–205. 10.1080/10502556.2017.1300010.

[jmft70165-bib-0016] Hawkins, A. J. , A. M. Galovan , S. M. Harris , and E. Fawcett . 2017. “What Are They Thinking? A National‐Sample Study of Stability and Change in Divorce Ideation.” Family Process 56, no. 3: 757–772. 10.1111/famp.12261. 28623842

[jmft70165-bib-0017] Joel, S. , S. C. E. Stanton , E. Page‐Gould , and G. MacDonald . 2021. “One Foot out the Door: Stay/Leave Ambivalence Predicts Day‐to‐Day Fluctuations in Commitment and Intentions to End the Relationship.” European Journal of Social Psychology 51, no. 2: 294–312. 10.1002/ejsp.2739.

[jmft70165-bib-0025] Johnson, L. N. , R. B. Miller, A. B. Bradford, and S. R. Anderson. 2017. “The Marriage and Family Therapy Practice Research Network (MFT‐PRN): Creating a More Perfect Union Between Practice and Research.” Journal of Marital and Family Therapy 43, no. 4: 561–572. 10.1111/jmft.12238. 28426921

[jmft70165-bib-0018] Kamp Dush, C. M. , W. D. Manning , M. N. Berrigan , et al. 2023. “National Couples' Health and Time Study: Sample, Design, and Weighting.” Population Research and Policy Review 42, no. 4: 62. 10.1007/s11113-023-09799-7. 37859760 PMC10586714

[jmft70165-bib-0020] Nylund, K. L. , T. Asparouhov , and B. O. Muthén . 2007. “Deciding on the Number of Classes in Latent Class Analysis and Growth Mixture Modeling: A Monte Carlo Simulation Study.” Structural Equation Modeling: A Multidisciplinary Journal 14: 535–569. 10.1080/10705510701575396.

[jmft70165-bib-0021] Nylund‐Gibson, K. , and A. Y. Choi . 2018. “Ten Frequently Asked Questions about Latent Class Analysis.” Translational Issues in Psychological Science 4, no. 4: 440–461. 10.1037/tps0000176.

[jmft70165-bib-0022] Owen, J. , G. Rhoades , B. Shuck , et al. 2014. “Commitment Uncertainty: A Theoretical Overview.” Couple and Family Psychology: Research and Practice 3, no. 4: 207–219. 10.1037/cfp0000028.

[jmft70165-bib-0023] Qin, H. , and L. Guo . 2026. “LCPA: A General Framework for Latent Classify and Profile Analysis. R package version 1.0.1.” https://CRAN.R-project.org/package=LCPA.

